# COPD awareness among the Syrian community: population-based study

**DOI:** 10.1038/s41598-023-32612-6

**Published:** 2023-04-12

**Authors:** Rana Hadakie, Ameer Kakaje, Khalil Al Kwatly, Shaden Haddad

**Affiliations:** 1 Department of Biochemistry and Microbiology, Faculty of Pharmacy, Damascus University, Damascus, Syria; 2 Faculty of Medicine, Damascus University, Damascus, Syria; 3 University Hospital Geelong, Barwon Health, Victoria, Australia

**Keywords:** Respiratory tract diseases, Health care

## Abstract

Chronic obstructive pulmonary disease (COPD) is a common disease and among the top causes of mortality worldwide but can be prevented and treated. This study aims to estimate the awareness of COPD among the Syrian population. A cross-sectional anonymous self-administered online survey was conducted by using Google Forms on Social Media platforms. The questionnaire included demographic, smoke-related and COPD-related questions. This study included 1607 participants with 930 (57.8%) females, 40% aging 21–25 years old, more than 90% being university students/graduates and 67.8% living in cities. Around half were either active smokers or had second-hand smoke exposure. After excluding participants in health-related fields who were 950 participants, only 25.4% of the remaining had ever heard of the term COPD. Knowing about COPD was not associated with reported smoking habits. No significant differences in awareness were seen between city and countryside dwellers, governate groups, genders, or age groups. Being in a health-related field was a major factor of being aware of COPD. COPD awareness in Syria is low, even amongst the well-educated group. Moreover, COPD risk factors of smoking and exposure to indoor and outdoor pollutants are common. Raising awareness is crucial in the Syrian community as COPD is highly prevalence.

## Introduction

Chronic obstructive pulmonary disease (COPD) is a common condition that has a high prevalence worldwide but it is preventable and can be managed^1^. It manifests as a partially reversible airflow limitation due to chronic inflammation that most commonly occurs due to exposure to particles and gases, mostly from cigarettes. It is often associated with symptoms of shortness of breath, mainly on exertion, chronic cough, and chronic sputum production^1–3^. In addition to cigarette smoking, COPD may arise from certain occupational or environmental exposures such as coal mining^4^. Pulmonary function tests (PFT) are essential to document airflow obstruction and confirm COPD diagnosis. Post bronchodilator airflow obstruction is defined as a ratio of forced expiratory volume in the first second to the forced vital capacity (FEV1/FVC) below 0.7^1,4^.

**Figure 1:**
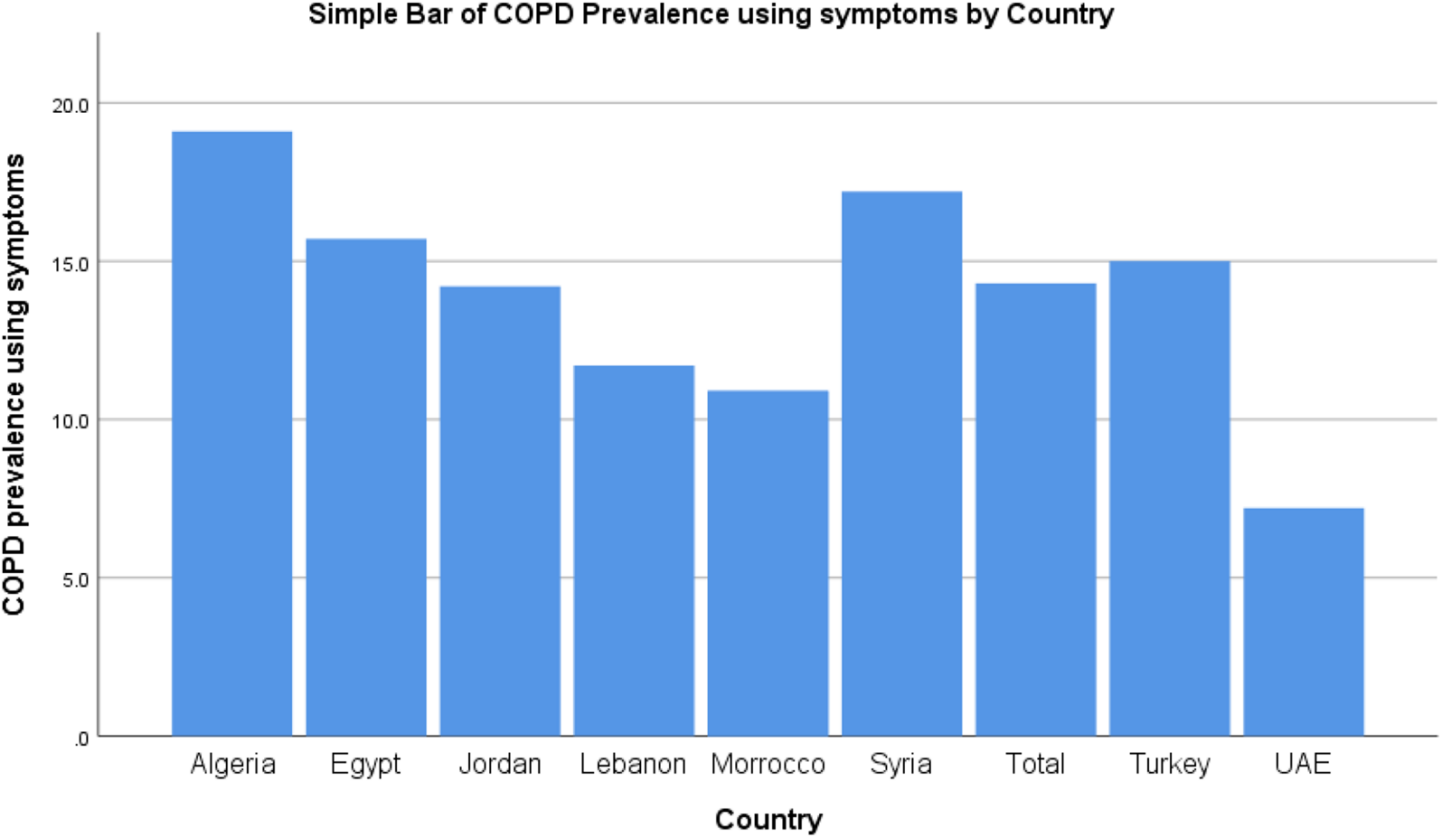
showing prevalence of COPD in various countries in the Middle East and Northern Africa using persistent symptoms of coughing or/and shortness of breath, numbers were cited from^7^.

COPD represents a major health concern and ranks as the 3rd leading cause of mortality globally, killing 3.23 million people in 2019, which was approximately 20% of the total deaths occurred in high-income countries^5^. COPD prevalence varies from one country to another^1^. Unfortunately, Syria is estimated to have one of the highest COPD prevalence among the Middle Eastern countries as it reached 17.2% compared to 14.3% in other countries in the Middle East and North Africa, adjusted by age and gender using symptoms of persistent coughing or breathlessness^6,7^ (Fig. 1). COPD is often underdiagnosed until it reaches moderate or severe stages or when the patient is hospitalized due to disease exacerbation^8^.

Poor public awareness about COPD and its symptoms is a major concern^9^. Research papers in numerous countries studied the level of knowledge related to COPD among the population and emphasised on the importance of increasing awareness^10–13^, especially to help increase smoking cessation and prevent smoking initiation. This is particularly important as governments around the world spend billions combat COPD and its complications, which is particularly important in low-income countries due to the high burden of COPD^6^. This study aimed to measure the knowledge related to COPD in Syrian population in light of its high prevalence^6^ and the high prevalence of tobacco and shisha smoking in Syria^14^.

## Methods

A cross-sectional anonymous self-administered online survey was conducted between 9 and 26 August 2021 among Syrian adults (age ≥ 18) through various social media platforms (WhatsApp and Facebook). It was posted daily and assured confidentiality on all participants. People had access to the link of the survey 24/7 and can access it using any device. The survey’s first page was confidentiality statement, explain the goals of the research and the reasons for the questionnaire.

The survey was originally developed following reviewing relevant literature, designed using the Google forms tool, and consists of 24 questions that were completed within 5 min. The questionnaire was split into several sections which were: demographic characteristics such as age, gender, education level, smoking habits, and basic knowledge about COPD such as its prevalence, definition, symptoms, and diagnostic test.

We confirm that all methods were performed in accordance with the relevant guidelines and regulations and in accordance with the Declaration of Helsinki.

All variables are presented as frequency and percentages. Chi-square test was used to determine statistical significance between proportions and differences were statistically significant when p values were ≤ 0.05. SPSS version 25 was used to analyse the data.

### Ethics approval and consent to participate

Online informed consent was taken before proceeding with the survey for participating in the research, and for using and publishing the data. We ensure confidentiality was maintained and asked no questions that might reveal any individual’s identity. No subjects were under the age of 18 years. Our study protocol and ethical aspects were reviewed and approved by Damascus University Faculty of Pharmacy, Damascus, Syria.

## Results

### Demographic characteristics

A total of 1619 Syrian adults started the survey, 1607 of them completed it fully, with 930 (57.8%) being females, and about 40% were in the age range of 21–25 years. In our study, 950 (59.1%) participants were either health-related field university students or graduates. Overall, 703 (43.7%) were university graduates. A total of 657 (40.9%) of respondents were of non-medical background and 67.8% of the total were living in Syrian cities. Demographics of the participants are shown in (Table 1).

**Table 1:** Demographic characteristics of the sample.

Characteristics	Non-medical background		Medical background	
	N	%	N	%
Gender				
Male	280	42.62	397	41.79
Female	377	57.38	553	58.21
Age (years)				
18 – 20	81	12.33	121	12.74
21 – 25	170	25.88	455	47.89
26 – 30	181	27.55	184	19.37
More than 31	225	34.25	190	20.01
Governorate				
Damascus	217	33.85	347	36.88
Damascus countryside	146	22.78	114	12.11
Aleppo	64	9.98	115	12.22
Homs	51	7.96	82	8.71
Hama	32	4.99	41	4.36
Lattakia	51	7.96	60	6.38
Tartus	27	4.21	56	5.95
Other	53	8.26	126	13.4
Place of residence				
City	415	63.07	676	71.08
Countryside	243	36.93	274	28.81
Education				
Non-Medical student	267	40.64	–	–
Non-medical graduates	276	42.01	–	–
High school diploma or less	114	17.35	–	–
Medical student	–	–	524	55.16
Medical graduates	–	–	426	44.84

### Smoking habits

Over half of participants (50.4%) had never smoked, whereas about 23% were current smokers. Characteristics of smoking are displayed in (Table 2). When using chi-square, no significant difference was found of smoking habits when comparing participants who have heard of COPD before or not (*p* = 0.810).

**Table 2:** Smoking habits of the participants.

Smoking habits	Non-medical background		Medical background	
	N	%	N	%
Smoking status				
Never smoked	297	54	513	54
Current smoker	178	22.54	187	22.54
Previous smoker (stop smoking for more than a month)	32	3.58	34	3.58
Second-hand smoker	150	19.68	22.74	19.68
Questions for only current smokers				
Smoking period (years)				
Less than a year	17	9.55	21	11.23
1–5	57	32.02	82	43.85
06-10	56	31.46	50	26.74
More than 10 years	48	26.97	34	18.18
Cigarettes or shisha				
Cigarettes	95	53.37	94	50.27
Shisha	55	30.9	70	37.43
Both	28	15.73	23	12.3
Cigarettes per day				
Less than 10	76	42.7	97	51.87
10–20	76	42.7	64	34.22
21–40	24	13.48	22	11.76
More than 40	2	1.12	4	2.14

### Knowledge about COPD

To minimise the effect of being in medical field on COPD-related knowledge as COPD is a part of their education, the sample was split into two groups: respondents who worked/studied in a medical-related field and those who were not. the main results in this study was related to participants that were not in health-related field.

In that group, only 167 (25.4%) had heard of COPD. There was no significant difference in participants who knew what COPD was when comparing genders and living in the city or the countryside for both samples, (p value was 0.185 for place of living and *p* = 0.323 for gender for the non-medical sample). When comparing age and governorates for the entire sample, there were insignificant differences *p* = 0.261 and *p* = 0.406 respectively (Table 3).

**Table 3:** Main risk factors and causes of COPD among people who have heard of COPD in medical- related sample.

	Non-medical background		Medical background	
	N	%	N	%
Have you ever heard about COPD?				
Yes	167	25.4	829	87.26
If the answer is Yes, answer these questions				
Main organ affected?				
Lungs	155	92.8	750	90.47
Do not know	11	6.6	0	0
Another organ	1	0.6	79	9.53
What do you think COPD is?				
Chronic Bronchitis and emphysema	120	71.9	574	69.24
Asthma	13	7.8	39	4.7
Cancer	1	0.6	3	0.36
Pulmonary fibrosis	33	19.8	68	9.89
Do you think that COPD is common?				
Yes	98	58.7	571	68.88
How to properly diagnose it?				
Pulmonary function test/spirometry	119	71.3	687	82.87
Symptoms	24	14.4	99	11.94
Other ways/I do not know	24	14.4	43	5.19
Do you believe it is life threatening?				
Yes	85	50.9	515	62.12
Do you believe it is treatable?				
Yes	79	47.3	166	20.02
What do you believe the main cause is?				
Smoking	131	78.4	713	86
Do not know	22	13.2	18	2.17
Other causes	14	8.4	98	11.82
What do you believe the main symptoms of COPD?				
Stated respiratory symptoms including dyspnea, chronic cough, and wheezing	138	82.6	829	100
Don’t know/stated other symptoms	29	17.4	0	0

## Discussion

To the best of our knowledge, this is the first study that has evaluated public awareness of COPD in the Syrian population. Although the majority of our participants were university students/graduate, the awareness of COPD was only around 25% in non-medical participants, which reflected low awareness. Among participants who stated they had heard of COPD before in our sample, there were around 40% of them who were not aware that COPD was common worldwide in our study and did not realize COPD was prevalent in Syria. Furthermore, when excluding health-related field population, awareness was even lower and did not significantly differ with governorate, living in the city or the countryside, gender, smoking, or age. The factor associated with increased awareness was working/studying in a health-related field.

COPD awareness in our study was different from other reported results from Slovenia (50%)^15^, Singapore where (35%) had ever heard of the term COPD^10^, Spain where only 17% spontaneously recognised COPD term^16^, France where 8% of participants were able to identify the COPD acronym^17^, and India where they had the lowest level of awareness about COPD as only 0.9 had heard the word COPD^11^. Our result may be explained by our studied group having higher socioeconomical status than the general population.

Among people who stated they have heard of the term COPD, smoking was stated by around 80% as the main risk factor for COPD. However, this was not associated with respondents reported smoking habits. A study in Turkey found that around half of the respondents chose tobacco smoking as the main risk factor of COPD^13^. Furthermore, a previous study in Syria found that 37.9% of participants were tobacco smokers, including smoking shisha, or/and cigarette^18^. This emphasises on the need to increase public awareness regarding harmful impacts of smoking on health between Syrians.

About three quarters of those who had heard about COPD were aware that COPD refers to chronic bronchitis or/and emphysema. One study in Singapore mentioned that only 10% of its participants were aware of what COPD acronym referred to^10^. In our study, respiratory symptoms were the most common reported symptoms. In the Slovenian study, dyspnoea also was the most knowledgeable symptom of COPD followed by cough^15^.

Around three quarters of those who stated that they know about COPD were aware that PFT was necessary to diagnose COPD, and only 50–60% stated that COPD could be a lethal disease, which showed the necessity to increase the knowledge about the danger of COPD on health.

In the Syrian community many COPD risk factors are prevalent, mainly high smoking rates found by our study and others which is highly prevalent in Syria and nearby countries^18,19^, and this was despite the rising beliefs against smoking after COVID-19 pandemic^20^. Second-hand smoke exposure was also common in our study which is also a risk factor for COPD.

Biofuel was found to be a risk factor of COPD^21^. One study found that around 3 billion people worldwide use biofuel, mainly in developing countries^22^.Biofuel is very common in Syria as people are forced to use it due to the deteriorating economy.

Air quality, either indoors or outdoors, is also an important factor in developing COPD as many pollutant can increase the risk of developing COPD^23^ and Syria has heavy air pollution, mainly in major cities.

Our finding indicated that there is an urgent need for enhancing levels of public awareness related to COPD in the Syrian community, mainly in participants who were not involved in health-related fields. Increasing awareness about COPD in this population is essential for both an early diagnosis of COPD and managing it, especially with the high prevalence rate of COPD in the Syrian population compared to the other regions and was more common amongst females^6,6^ (Fig. 1). Increasing awareness could be accomplished through possible interventions such as using social media in providing information related to COPD by health authorities. Social media platforms provide information that are easily accessible and could reach a great number of people. Running awareness campaigns in targeted groups could be effective in increasing public COPD awareness.

This study came with limitations that affect the generalizability of the results, mainly two; using online methods limited the targeted population and having high educational levels and high rates of respondents in health- care related work or education in this study indicate that the estimated awareness of COPD in Syrian population in our study was probably overestimated as the majority were young and university students or graduates. We attempted to minimise the effect by dividing the sample into two groups and basing the results mainly on respondents that were not studying/working in health-related field which were medicine, pharmacy, dentistry and nursery to reduce the effect of medical background on results as possible.

Another limitation was regarding using convenient sampling method, we have used this method as gathering samples from the entire community was not possible and we aimed to be as wide as possible in the community to test their knowledge about COPD.

Some missing data in some questions, but the response rate for each question exceeded 95%. No accurate indicated of social-economy status could be used due to the cultural barrier that prevented us from asking about monthly income of the family and difficulty to standardise the results with international standards.

In conclusion, there is a lack of awareness of COPD in the Syrian population. Increasing awareness is an emergent priority to help COPD diagnosis and management. COPD and its risk factors are very common in Syria, mainly air pollutants exposure from either actively smoking or indoors/outdoors exposure to particles. There was no significant difference of awareness in non-health related participants when comparing different groups such as genders, ages, governorates, living in the city or countryside and smoking habits.

## Data Availability

The data can be made available upon reasonable request from the corresponding author.

## Abbreviations

COPD: Chronic obstructive pulmonary disease
FEV1: Forced expiratory volume in the first second
FVC: Forced vital capacity
PFT: Pulmonary function test
SPSS: Statistical package for the social sciences
